# A PTX3/TLR4 axis sustains sonic hedgehog-medulloblastoma growth and defines a new therapeutic vulnerability

**DOI:** 10.1093/neuonc/noag167

**Published:** 2026-07-23

**Authors:** Serena Filiberti, Camilla Tavani, Elena Somenza, Nicolas Tassone, Davide Capoferri, Chiara Romani, Arianna Giacomini, Elisabetta Grillo, Marco Ritelli, Tiziana Annese, Antonio d’Amati, Martina Canton, Elena Mariotto, Roberta Bortolozzi, Giampietro Viola, Andrei Turtoi, Pietro Luigi Poliani, Marta Turati, Roberto Ronca

**Affiliations:** Department of Molecular and Translational Medicine, University of Brescia, Brescia, Italy; Department of Molecular and Translational Medicine, University of Brescia, Brescia, Italy; Department of Molecular and Translational Medicine, University of Brescia, Brescia, Italy; Department of Molecular and Translational Medicine, University of Brescia, Brescia, Italy; Department of Molecular and Translational Medicine, University of Brescia, Brescia, Italy; Department of Medical and Surgical Specialties, Radiological Sciences and Public Health, University of Brescia and ASST Spedali Civili di Brescia, Brescia, Italy; Department of Molecular and Translational Medicine, University of Brescia, Brescia, Italy; Department of Molecular and Translational Medicine, University of Brescia, Brescia, Italy; Department of Molecular and Translational Medicine, University of Brescia, Brescia, Italy; Department of Medicine and Surgery, LUM University, Casamassima (Bari), Italy; Department of Medicine and Surgery, LUM University, Casamassima (Bari), Italy; Fondazione Policlinico Universitario Agostino Gemelli IRCCS, Università Cattolica del Sacro Cuore, Rome, Italy; Laboratory of Pediatric Hematology-Oncology Department of Women’s and Children’s Health, University of Padova, Padova, Italy; Istituto di Ricerca Pediatrica (IRP), Padova, Italy; Laboratory of Pediatric Hematology-Oncology Department of Women’s and Children’s Health, University of Padova, Padova, Italy; Istituto di Ricerca Pediatrica (IRP), Padova, Italy; Istituto di Ricerca Pediatrica (IRP), Padova, Italy; Department of Pharmaceutical and Pharmacological Sciences, Section of Pharmacology, University of Padova, Padova, Italy; Laboratory of Pediatric Hematology-Oncology Department of Women’s and Children’s Health, University of Padova, Padova, Italy; Istituto di Ricerca Pediatrica (IRP), Padova, Italy; Institut de Recherche en Cancérologie de Montpellier, Université de Montpellier, INSERM, Montpellier, France; Department of Molecular and Translational Medicine, University of Brescia, Brescia, Italy; Department of Molecular and Translational Medicine, University of Brescia, Brescia, Italy

**Keywords:** medulloblastoma, pediatric tumor, PTX3, sonic hedgehog medulloblastoma, TLR4 signaling pathway

## Abstract

**Background:**

Sonic hedgehog-medulloblastoma (SHH-MB) represents a biologically diverse and clinically challenging subgroup, especially in high-risk variants characterized by metastatic dissemination. Despite progress in molecular stratification, current treatments rely on intensive multimodal regimens that are associated with substantial long-term neurotoxicity. Identifying novel oncogenic mechanisms that can be therapeutically exploited is therefore a critical priority.

**Methods:**

We combined transcriptomic analyses of patient datasets with immunohistochemistry, RNAscope, and protein profiling to evaluate PTX3 expression in MB subgroups. Functional studies were performed in SHH-MB cell lines following PTX3 knockdown or knockout. Effects on proliferation, migration, and angiogenesis were assessed through multiple *in vitro* assays. *In vivo* relevance was tested using subcutaneous and orthotopic xenograft models. Mechanistic insights were obtained through phospho-kinase arrays, Western blotting, and GSEA. A high-throughput drug screen and combination studies with specific inhibitors and standard chemotherapy (vincristine/cisplatin/cyclophosphamide; VCC) were performed to assess therapeutic potential.

**Results:**

Our results reveal that PTX3 was strongly enriched in SHH-MB subgroup, where it supported proliferation, motility, and angiogenesis. Mechanistically, PTX3 activated a TLR4-dependent IRAK1/PI3K-Akt/GSK-3/β-catenin signaling cascade. Genetic downregulation or pharmacologic blockade of PTX3-TLR4 significantly reduced tumor growth and angiogenesis *in vivo*. Moreover, the TLR4 inhibitor TAK-242 markedly impacted SHH-MB growth *in vitro*, and combination therapy of TAK-242 and VCC produced additive/synergistic effects *in vitro* and significantly prolonged survival in orthotopic SHH-MB-bearing mice.

**Conclusions:**

Our findings identify PTX3-TLR4 signaling as key oncogenic driver and a promising therapeutic ­vulnerability in SHH-MB. Targeting this pathway enhances the efficacy of standard therapy and represents a rationale for mechanism-based combination strategies.

## Graphical Abstract

**Graphical Abstract:**
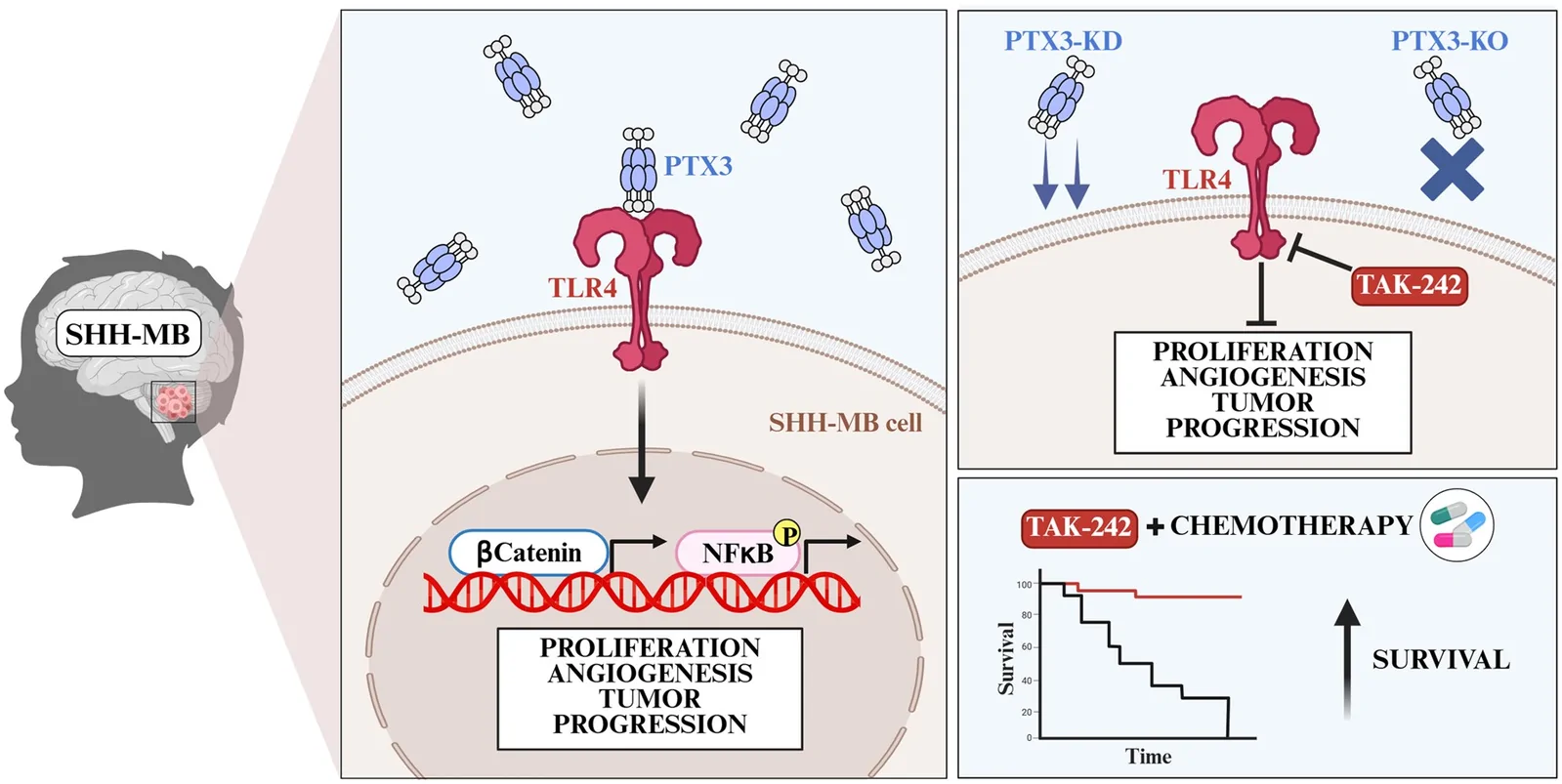

Key Points

- PTX3 is overexpressed in SHH-MB and drives tumor proliferation, migration, and angiogenesis.
- PTX3 activates a TLR4-dependent IRAK1/AKT/β-catenin oncogenic signaling program.
- Targeting PTX3-TLR4 enhances chemotherapy efficacy and prolongs survival in SHH-MB.

Importance of the Study

Medulloblastoma (MB), particularly the sonic hedgehog (SHH) subgroup, remains a major clinical challenge due to its molecular heterogeneity, high-risk variants, and the substantial toxicity of current treatments. Identifying actionable pathways by driving SHH-MB aggressiveness is therefore critical for improving therapeutic outcomes. Here, we uncover Pentraxin-3 (PTX3) as a previously unrecognized, SHH-enriched oncogenic mediator that promotes proliferation, migration, clonogenicity, and angiogenesis. Mechanistically, we demonstrate that PTX3 activates a TLR4/IRAK1/PI3K-Akt/GSK-3/β-catenin signaling cascade, establishing a key regulatory axis essential for SHH-MB growth. Importantly, genetic silencing or pharmacological blockade of PTX3-TLR4 profoundly impairs tumor progression *in vitro* and *in vivo*. We further show that combining the TLR4 inhibitor TAK-242 with standard chemotherapeutic agents yields additive or synergistic antitumor effects and significantly prolongs survival in an orthotopic SHH-MB model.

These findings identify PTX3-TLR4 signaling as a mechanistic driver and a promising therapeutic vulnerability in SHH-MB, offering a rationale for pathway-oriented combination therapies.

Brain tumors represent the most common solid malignancies in children and the second-leading cause of cancer-related mortality in the pediatric population.^1^^,^^2^ Among central nervous system (CNS) tumors, medulloblastoma (MB) constitutes the most frequent malignant pediatric brain tumor, with annual incidence of MB of about 5 cases per 1 million individuals and peak onset between 3 and 9 years of age.^3^^,^^4^ Advances in molecular profiling have profoundly reshaped the biological and clinical understanding of MB, and the current WHO CNS classification integrates both histopathological and molecular features, reflecting the tumor’s intrinsic complexity, genomic diversity, and clinical variability.^5-7^ At molecular level, 4 major subgroups are recognized: WNT-activated, SHH-activated, and the non-WNT/non-SHH categories encompassing Group 3 and Group 4 tumors.^8^ These subgroups differ in cellular origin, driver alterations, age distribution, and prognosis, with survival rates ranging from less than 55% in high-risk forms to around 95% in more favorable subtypes.^9^ The SHH subgroup accounts for approximately 30% of MB cases, exhibits an intermediate prognosis with an overall survival rate around 75%, and outcomes are markedly worse in the presence of metastases or high-risk molecular alterations such as *TP53* mutations or *MYCN* amplifications.^7^^,^^10^ Current SHH-MB management relies on maximal safe resection followed by radiotherapy and chemotherapy.^11^^,^^12^ Although effective, these intensive treatments are associated with significant long-term neurocognitive and neurological sequelae.^13^^,^^14^ Consequently, the identification of novel molecular targets is essential to improve therapeutic outcomes while minimizing treatment-related toxicity.^15^

Long pentraxin-3 (PTX3) is a soluble pattern recognition receptor of the pentraxin family and a key mediator of innate immunity, inflammation, and pathogen recognition.^16^^,^^17^ PTX3 participates in various physiological and pathological processes, including cancer, where it regulates tumor growth, angiogenesis, metastasis, and immune modulation.^18^ Interestingly, PTX3 exerts context-dependent functions: it can act as an oncosuppressor by modulating complement-dependent inflammatory responses and sequestering tumor-promoting fibroblast growth factors (FGFs),^18-22^ yet it can also promote tumor progression. Pro-tumor activity of PTX3 has been documented in gastric, cervical, lung, and breast cancers, as well as in glioma, pancreatic, hepatocellular, and head and neck squamous cell carcinomas.^23-29^ In these settings, PTX3 secreted by tumor or inflammation-associated stromal cells enhances cell proliferation, invasion, epithelial to mesenchymal transition (EMT), macrophage recruitment, and metastatic behavior.

Within the CNS, PTX3 is predominantly associated with inflammatory responses and neurodegeneration.^30^ Under physiological conditions, PTX3 expression is low in brain tissue, however, it becomes markedly upregulated in neoplastic contexts, including glioma, meningioma, and neuroblastoma.^30^ Multiple lines of evidence support a tumor-promoting role for PTX3 in glioblastoma (GBM), where its increased expression enhances proliferation, migration, and invasiveness.^26^^,^^31^^,^^32^ PTX3 promotes the transcription and secretion of periostin (POSTN), activates the MAPK-ERK pathway, supports EMT-like programs, and contributes to maintaining the glioma stem cell phenotype.^33^ Additional data demonstrates PTX3 involvement in the tumor-promoting activity of sphingosine kinase 1 (SPHK1),^34^ its upregulation through the oncogenic SPOCD1 axis,^35^ and its ability to regulate ferritinophagy via protein arginine methyltransferase 1 (PRMT1).^36^ Moreover, PTX3 modulates macrophage recruitment and polarization toward inflammation-resolving phenotypes within the GBM microenvironment, enhancing CD68 and CD163 expression.^32^^,^^37^ A pro-tumor role has also recently been suggested in neuroblastoma.^38^ Despite the emerging literature supporting PTX3 as a tumor-promoting factor in CNS malignancies, its potential role in MB remains completely unexplored.

In this study, we report for the first time that PTX3 is preferentially expressed by tumor cells in SHH-MB, where it promotes proliferation, angiogenesis, and tumor growth. Mechanistically, we demonstrate that PTX3 exerts its pro-tumor activity through activation of TLR4 signaling, leading to β-Catenin and NF-κB activation. Notably, inhibition of the PTX3/TLR4 axis exhibits significant therapeutic efficacy in SHH-MB models, both as monotherapy and in combination with clinically relevant treatments.

## Methods

### Reagents and Cell Cultures

Human medulloblastoma cell lines DAOY (HTB-186), HD-MB03 (CVCL_S506), ONS-76 (CVCL_1624) and UW-228 (CVCL_8585) were obtained from American Type Culture Collection (ATCC). DAOY cells were grown in DMEM, supplemented with 10% fetal bovine serum (FBS), 2mM glutamine, and 1% penicillin/streptomycin. ONS-76 were grown in RPMI 1640 medium, supplemented with 10% FBS, 2mM glutamine, and 1% penicillin/streptomycin. UW-228 in DMEM/F12, supplemented with 10% FBS, 2mM glutamine and 1% penicillin/streptomycin. Murine L90 cells are Ptch1^+/−^ p53^−/−^ derived cells as described in,^39^ kindly provided by Dr. R. Galli (San Raffaele Scientific Institute, Milan) and grow in DMEM/F-12 supplemented with 2mM glutamine, B-27, penicillin/streptomycin, bFGF (20ng/mL) and EGF (20ng/mL). For silencing, cells were infected with lentiviral vector containing short-hairpin RNA (shRNA) targeting human PTX3 (TRCN0000436981 or TRCN0000430959) to generate PTX3-silenced (shPTX3) cells or a non-targeting/control sequence (SHC002V, Merck Millipore, Burlington, MA, USA) to generate control (shNT) cells. Transduced cells were selected with 1µg/mL puromycin. Cells were maintained at low passage, returning to original frozen stocks every 3 to 4 months, and tested regularly for Mycoplasma negativity. For PTX3 knockout, DAOY cells were infected with PTX3 CRISPR Virus Pool packaging of PTX3 CRISPR All-in-One Lentivirus (Set of 3 Targets, Human: GTGAACTTACGGTCCTCAGT, TTCCGAGTGCTCCTGACCGC, GCTGGCGCGTATGGAGGGCG; ABM Goods). Transduced cells were selected with 1µg/mL puromycin.

The TLR4 inhibitor TAK-242 was purchased from Selleckchem (Houston, TX, USA), CHIR-98014 (GSK-3 α/β inhibitor), and LY294002 (PI3K inhibitor) from MedChemExpress LLC (Monmouth Junction, NJ, USA), while VCC (Vincristine from Tocris Bioscience, Cat.No.12.57; Cisplatin from Santa Cruz Biotechnology, CAS 15663-27-1; Cyclophosphamide from Santa Cruz Biotechnology, sc-206885B).

### In Vitro Assays

#### Cell Proliferation

Cells were seeded (4 × 10^3^) in 48-well culture plates in complete medium. After 24hrs, cells were starved at 1%FBS and detached at 72 hours and counted using the MACSQuant Analyzer (Miltenyi Biotec, Bergisch Gladbach, Germany).

#### Clonogenic Assay

Cells (250) were seeded in 6-well culture plates and incubated in complete growth medium until visible colonies were formed. Then, the supernatant was removed and cells stained with 0.1% crystal violet/20% methanol. Plates were photographed to count formed colonies using the ImageJ software. Finally, crystal violet staining was solubilized with 1% SDS solution to measure absorbance at 595 nm.

#### Wound-Healing Assay

Confluent cells were scraped with a 200 µL tip to obtain a 2-mm-thick denuded area. After 12 hours, wounded monolayers were photographed and the width of the wounds was measured in 3-independent sites per group.

### qPCR Analysis

Total RNA was extracted using QIAzol reagent, treated with DNAse, and 2 μg of total RNA were retro-transcribed with MMLV-RT using random hexaprimers, cDNA was analyzed by quantitative PCR using primers specific for human or murine PTX3 (hPTX3: Forward primer: 5′-CATCTCCTTGCGATTCTGTTTTG-3′; reverse primer: 5′-CCCATTCCGAGTGCTCCTGA-3′). Housekeeping gene human GAPDH was detected for normalization (hGAPDH: Forward primer: 5′-GAAGGTCGGAGTCAACGGATT-3′; reverse primer: 5′-TGACGGTGCCATGGAATTTG-3′).

### Human Phospho-Protein Proteome Profiler Array

shPTX3 and shNT DAOY cells were seeded at a density of 750,000 cells per well in complete medium supplemented with 10% FBS. After 24 hours, the medium was replaced with serum-free medium (0% FBS) to induce serum starvation for 30 hours. Following serum starvation, cells were lysed, and the total protein concentration was determined using a standard protein quantification assay (Bradford). A total of 600 µg of protein lysate from shPTX3 and shNT DAOY cells was incubated with the human phospho-kinase array kit (R&D Systems) according to manufacturer’s instructions. The chemiluminescent signal was acquired and analyzed using the ImageJ software.

### Western Blot Analyses

Cells were lysed in NP-40 lysis buffer. Protein concentrations were determined using the Bradford protein assay (Bio-Rad Laboratories, Milano, Italy). Then, 30 μg protein/sample were separated by SDS-PAGE and blotted on a PVDF membrane. The following antibodies were used: anti-PTX3 (from B. Bottazzi, Humanitas Clinical Institute, Rozzano, Italy), anti-TLR4 (Bio-Rad), anti-phospho IRAK1 (T209; Abcam), anti-phospho AKT(S473); Cell Signaling Technology, MA, USA), anti-phospho p65 (Ser311; Santa Cruz Biotechnology, CA, USA), anti-p65 (Santa Cruz Biotechnology), anti-phospho PI3K p85 (Tyr458)/p55 Cell Signaling, anti-Akt (C67E7, Cell Signaling), anti-phospho GSK-3a/ß (S21/9; Cell Signaling), anti-ß-Catenin (Cell Signaling). To normalize the amount of loaded proteins, all blots were probed with anti-βactin (Sigma-Aldrich), anti-α-tubulin (Sigma-Aldrich), anti-GAPDH (Santa Cruz Biotechnology). Chemiluminescent signal was acquired by ChemiDoc Imaging System (Bio-Rad) and analyzed using the ImageJ software.

### Genome-Wide Expression Profiling

The ClariomD Assay Human was used to generate the expression profiles of shNT/shPTX3 DAOY cells using biological triplicates in each condition. Total RNA was extracted using TRIzol Reagent according to manufacturer’s instructions (Invitrogen, Waltham, MA, USA). RNA integrity and the purity of the treated cells were assessed using a Bioanalyzer (Agilent Technologies, Santa Clara, CA, USA). The microarray experiments were performed in accordance with the manufacturer’s instructions, starting from 100 ng of total RNA, using the GeneChip WT PLUS kit (ThermoFisher). After hybridization, the arrays were washed and stained using the GeneChip Fluidics Station 450, followed by scanning with the GeneChip Scanner 3000 7G used in conjunction with GeneChip Operation Software (ThermoFisher Scientific) to generate one CEL file per hybridized cDNA. After quality controls (QC), Analysis of variance (ANOVA) test was performed to assess the effects of PTX3 modulation on gene expression, comparing DAOY shNT vs DAOY shPTX3. To identify significantly enriched or depleted groups of genes involved in the same biological pathways, Gene Set Enrichment Analysis (GSEA) on genome-wide expression profiling (GEP) data was performed (http://software.broadinstitute.org/gsea/index.jsp). The dataset is available at https://www.ncbi.nlm.nih.gov/geo/query/acc.cgi? acc=GSE311435

### Human Angiogenesis Array

Cell Supernatants from DAOY shNT and DAOYshPTX3 cells were collected after 30 hours of culture in DMEM 0% FBS and concentrated with a 10kDa Amicon Ultra 15 centrifugal filter. Conditioned media were then incubated with the using the proteome profiler array Human Angiogenesis Array Kit (R&D Systems, Minneapolis, Canada) according to manufacturer’s instructions. Pixel densities were analyzed using the image analysis ImageJ software and normalized on reference spots.

### HUVECs 3D Sprouting Assay

HUVECs spheroids were prepared in 20% methylcellulose medium, embedded in fibrin gel and co-culture with PTX3-silenced cells. After 24 hours sprouts were counted and photographed.

### Chick Chorioallantoic Membrane Assay

Fertilized white Leghorn eggs were incubated at 37°C in a humidified incubator. At 4 days post-incubation, the shells were covered with a transparent adhesive tape and a small window cut with scissors; windows were then resealed with tape. At 11 days post-incubation, alginate beads containing the sample (2 × 10^4^ in 3µL PBS/egg: DAOY PTX3-KO and MOCK clones or DAOY shNT and shPTX3 cell) under test were placed on top of the chick chorioallantoic membrane (CAM) of fertilized White Leghorn chicken eggs. After 72 hrs, blood microvessels entering the implants within the focal plane of the CAM were counted in ovo at ×5 magnification using a STEMI SR stereomicroscope. The experiments were repeated at least twice with 7-10 eggs per group.

### High Throughput Screening

Drug screening was performed in collaboration with the University of Padova on DAOY shNT and DAOY shPTX3 cells. DAOY cells were seeded at 400 cells/well in 27μL/well of their complete growth medium in 384-well flat bottom plate (#3701 Corning, Corning, NY). The day after seeding, cells were treated with the compound library at the final concentration of 5 µM (in 0.05% final dimethyl sulfoxide/DMSO concentration). The high throughput screening (HTS) drug library consisted of 3533 compounds acquired from different commercial sources, as previously described.^40^ Each plate of the screening library contained 320 test compounds while internal negative (DMSO 0.05%) and positive (Benzethonium Chloride 75μM) controls were arranged in the external columns 1, 2, 23, and 24. After 72 h of treatment, a resazurin-based assay was performed to assess cell viability. To ensure treatment reproducibility, all procedures, including cell seeding, drug dilutions, cell treatment, and application of resazurin solution, were carried out through the Microlab STAR 96-CORE (Hamilton, Bonaduz, Switzerland) liquid handler.

The Z-prime factor (Z’) was monitored as HTS QC, and only plates with Z’>0.4 were analyzed to ensure robustness of results. Raw data were normalized using the median calculated among all sample wells, excluding reference wells containing controls. Drugs exhibiting high selectivity for shNT cell line (shNT cell death >70%) while having minimal activity on shPTX3#2 (shPTX3#2 cell death <30%) were selected as HTS hits. For details regarding the library and procedures refer to.^40^

### In Vivo Studies

Animal Experiments were performed according to the Italian laws (D.L. 116/92 and following additions) that enforce the EU 86/109 Directive and were approved by the local animal ethics committee (OPBA, Organismo Preposto al Benessere degli Animali, Università degli Studi di Brescia, Italy).

#### Subcutaneous Xenografts

Seven-week-old NOD/Scid mice were injected subcutaneously into the with 3 × 10^6^ human MB cells (DAOY PTX3-KO and MOCK clones or ONS-76 shNT and shPTX3 cell populations or L90 shNT and shPTX3 cells). Tumors were measured with calliper, and the volume was calculated according to the formula V = (D × d2)/2, where D and d are the major and minor perpendicular tumor diameters, respectively. At the end of the experimental procedure, tumors were surgically removed, weighed and paraffin embedded for immunohistochemical analysis.

#### Orthotopic Xenograft

Luciferase expressing ONS-76 cells (ONS-76-luc) were stereotactically injected (2 × 10^5^ in 3µL PBS) into the cerebellum of NOD/Scid mice 6.6 mm posterior to bregma, 1 mm lateral to the midline, and 2 mm ventral to the skull surface. Tumor progression was monitored by bioluminescence (BLI) imaging using an IVIS Lumina III apparatus (Revvity) after receiving an i.p. injection with of D-Luciferin, and analysis of the BLI was performed using the Living Image Software. At the end of the experimental procedures, mice were sacrificed and brains collected for further analyses. Treatments were performed i.p. according to the schedule illustrated in the manuscript with TAK-242 treatment (3 mg/Kg) and VCC (cisplatin 2.5 mg/kg + vincristine 0.1mg/kg on the first day and cyclophosphamide 75 mg/kg on the second day).

### Immunohistochemical and RNAscope Analyses

For IHC on tumor xenograft samples, formalin-fixed, paraffin-embedded samples were sectioned at a thickness of 3 µm, dewaxed, hydrated, and stained with hematoxylin and eosin (H&E) or processed for immunohistochemistry with rabbit anti-human PTX3 (from B. Bottazzi, Humanitas, Rozzano, Italy), anti-Ki67 antibody (ab15580, Abcam), anti-CD31 antibody (anti-mouse CD31/DIA-310, SZ31, Dianova), anti-phospho IRAK1 (T209; Abcam), or anti-phospho AKT (S473; Cell Signaling Technology). Positive signal was revealed by 3,3'-diaminibenzidine (Roche) staining. Sections were finally counterstained with Carazzi’s hematoxylin before analysis by light microscopy. Images were acquired with the automatic high-resolution scanner Aperio System (Leica Biosystems), and image analysis was carried out using the open-source ImageJ software.

For RNAscope on patients’ samples, in situ hybridization was performed on FFPE biopsies using RNAscope 2.5 HD Reagent Kit (RED 322360, Advanced Cell Diagnostics [ACD], Hayward, CA). The sections were covered with the Hs-PTX3 probe (ref. 517611) and then processed for Synaptophysin protein evaluation by immunohistochemistry. Sections were scanned using the whole-slide morphometric analysis scanning platform Aperio ScanScope CS (Leica Biosystems) at the maximum magnification available (40×) and stored as high-resolution digital images.

### Statistical Analyses

Statistical analyses were performed using Prism 8 (GraphPad Software). Student’s *t* test for unpaired data (2-tailed) was used to test the probability of significant differences between two groups of samples. For more than two groups of samples, data were analyzed with a 1-way analysis of variance and corrected by the Bonferroni multiple comparison test. Tumor volume data were analyzed with a 2-way analysis of variance and corrected by the Bonferroni test. Differences were considered significant when *P *< .05 unless otherwise specified.

## Results

### PTX3 Is Highly Expressed in SHH-MB

The expression of *PTX3* was analyzed in tumor samples from MB patients using R2 Genomics Analysis and Visualization Platform (RRID: SCR_025770; https://hgserver1.amc.nl/cgi-bin/r2/main.cgi), and the publicly available Cavalli dataset (GSE85217). Interestingly, PTX3 is highly expressed in MB samples if compared with normal cerebellum (Supplementary Figure S1A and B). Moreover, an in-depth analysis into the MB subtypes revealed that PTX3 mRNA levels are significantly increased in SHH-MB if compared with other groups (Figure 1A, Supplementary Figures S1C and D and S2). To confirm this observation, we performed immunohistochemistry analyses (IHC) on a cohort of 22 patients’ samples representative of the MB groups and confirmed that PTX3 is preferentially overexpressed in the SHH-MB samples (Figure 1B and C and Supplementary Figure S3). Given that PTX3 is a secreted soluble protein, we sought to determine its cellular source within the tumor microenvironment. RNAscope analysis performed on patient-derived MB samples demonstrated a pronounced enrichment of PTX3 mRNA in SHH-MB tissues, with signal spatially colocalizing with Synaptophysin-positive tumor cells, indicating tumor-intrinsic PTX3 production (Figure 1D and E).

**Figure 1.**
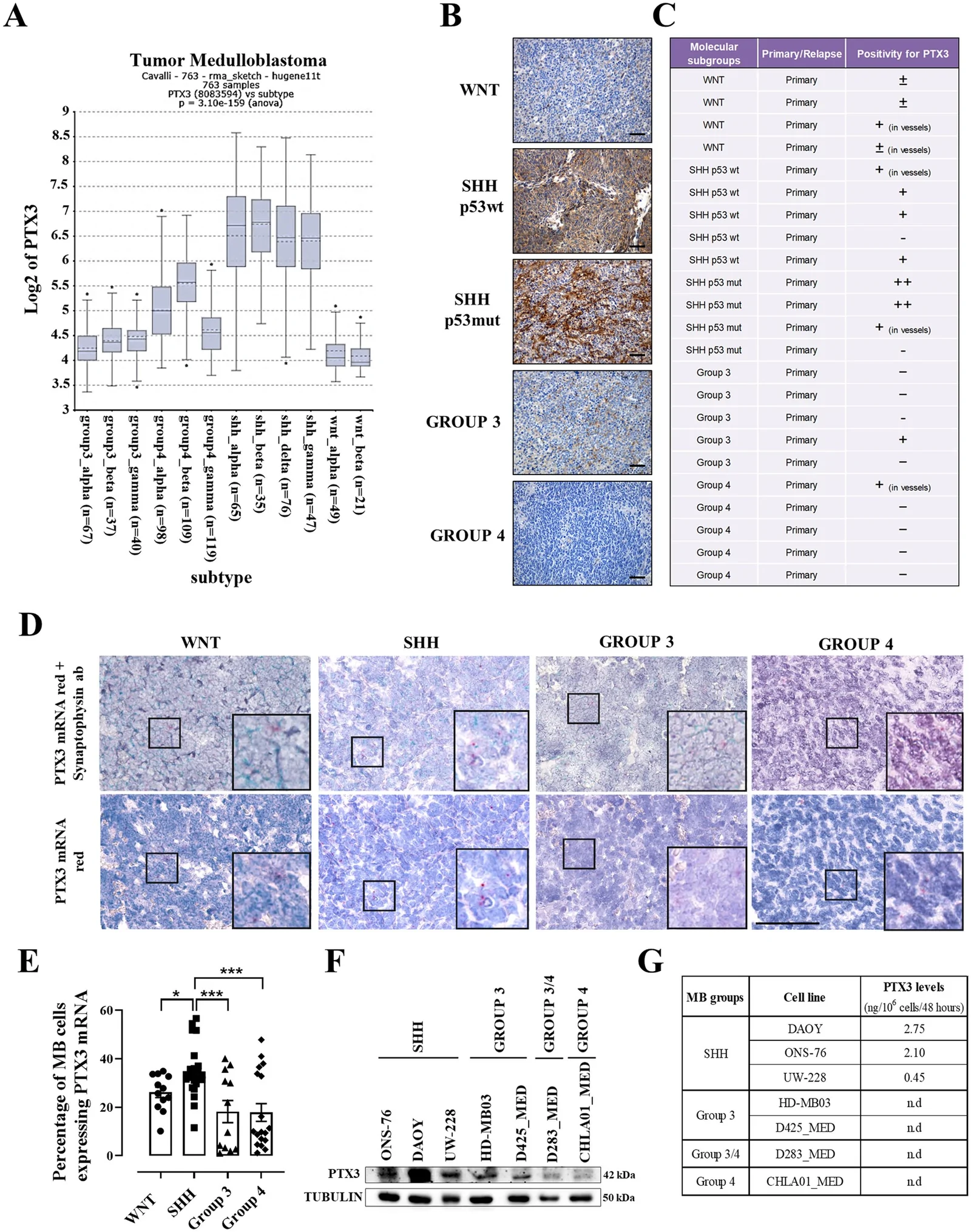
PTX3 expression in human MB. (A) PTX3 expression in MB subgroups by R2 Genomics Analysis and Visualization Platform (Cavalli dataset). (B) Representative pictures of immunohistochemistry for PTX3 on primary MB patient’s samples and (C) scoring table for PTX3 expression; scale bar 50 μm. (D) RNAscope images for PTX3 mRNA expression (arrows/red dots) and quantification (E) in WNT, SHH, Group 3 and Group 4 MB patient’s samples; scale bar 120 μm. (F) Western blot and ELISA (G) for PTX3 protein levels in human MB cells. Data are the mean ± SEM; n.d.= not detectable (ELISA detection limit 75pg/ml); *P < .05, **P < .01, ***P < .005.

In line with these results, Western blot analysis and ELISA quantification of human MB cell lines are representative of the different molecular groups (ONS-76, DAOY, UW-228, HD-MB03, D425Med, D283Med, CHLA-01-Med) confirmed elevated PTX3 protein levels specifically in SHH-MB models (Figure 1F and G).

Collectively, these findings demonstrate that PTX3 is preferentially and strongly expressed in the SHH-MB subgroup, suggesting a potential functional role in its biology.

### PTX3 Down-Modulation Impacts the Aggressiveness of SHH-MB Cells

To investigate the functional contribution of PTX3 to SHH-MB biology, 3 prototypic human SHH-MB cell lines, DAOY, ONS-76, and UW228, characterized by high endogenous PTX3 expression, were engineered for stable PTX3 down-modulation. Lentiviral transduction with two independent PTX3-targeting short-hairpin RNAs (shRNAs) resulted in efficient PTX3 silencing in all models compared with non-targeting controls (shNT) (Figure 2A-C and Supplementary Figure S4A-D).

**Figure 2.**
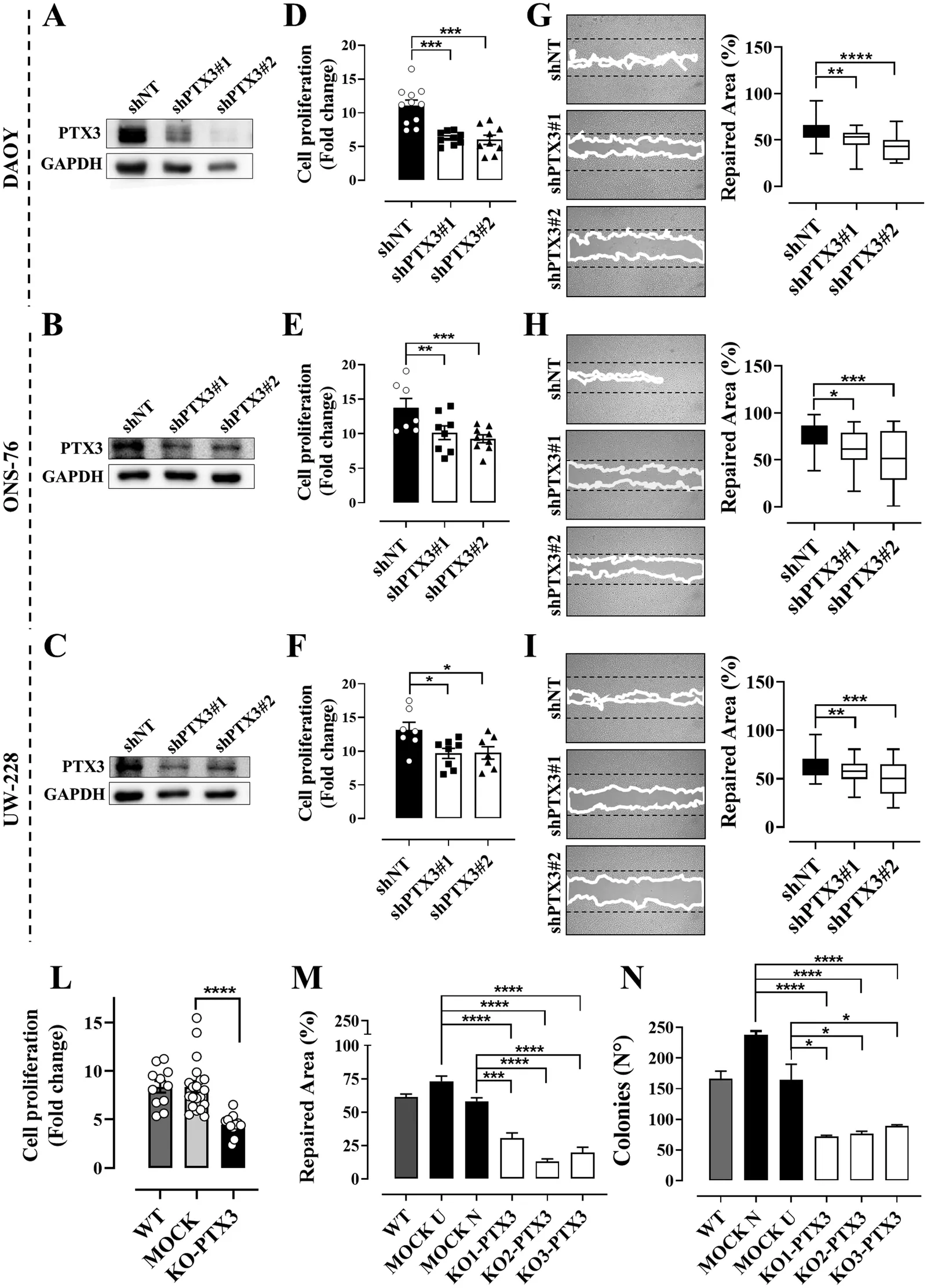
PTX3 downregulation and KO affect the biological features of SHH-MB cells. PTX3 silencing was verified by Western blot in (A) DAOY, (B) ONS-76 and (C) UW-228. Silenced (shPTX#1 and #2) and control (shNT) DAOY, ONS-76 and UW-228 cells were tested for their proliferation at 72 hours (D-F) and migratory properties in the wound healing assay (G-I). PTX3 Knock-out (KO) and control (MOCK) clones were generated for DAOY cells and clones were tested for proliferation (L), migratory properties in wound healing assay (M) and clonogenic capacity (N). Data are the mean ± SEM; *P < .05, **P < .01, ***P < .005, ****P < .001.

*In vitro* functional assays revealed that PTX3 knockdown significantly impaired cellular proliferation across all SHH-MB cell lines (Figure 2D-F). In addition, PTX3-silenced cells displayed a marked reduction in migratory ability, as assessed through wound-healing assays (Figure 2G-I). Consistently, comparative Gene Set Enrichment Analysis (GSEA) performed on DAOY shPTX3 cells demonstrated a trend in negative enrichment of gene programs associated with proliferation and migration (Supplementary Figure S5), supporting a PTX3-dependent regulation of these oncogenic processes.

To further corroborate these findings, we generated PTX3-knockout (PTX3-KO) DAOY clones using CRISPR/Cas9-mediated gene editing. Successful PTX3 ablation was confirmed by ELISA (Supplementary Figure S6A) and validated by Sanger sequencing and Next Generation Sequencing (NGS) (Supplementary Figure S6B). Three representative PTX3-KO clones, along with MOCK controls, were selected for additional characterization. In agreement with the knockdown data, PTX3-KO clones exhibited a significant reduction in proliferation, migration, and clonogenic capacity (Figure 2L-N and Supplementary Figure S7) relative to MOCK controls. Finally, in a representative MB Group 3 cell line (HD-MB03), in which PTX3 expression is intrinsically very low, PTX3 silencing produced no effects on proliferative activity (Supplementary Figure S8), further supporting the subgroup-specific relevance of PTX3 in SHH-MB biology. Altogether, these in vitro findings strongly support a functional role for PTX3 in sustaining key malignant traits of SHH-MB cells.

### *PTX3 Down-Modulation Impacts SHH-MB Tumor Growth* In Vivo

To further assess the tumor-promoting role of PTX3 in SHH-MB, PTX3-modified cells were evaluated *in vivo* for their tumor-forming capacity. PTX3-knockout (PTX3-KO) and control (MOCK) DAOY clones, along with wild-type cells, were injected subcutaneously into immunodeficient mice, and tumor growth was monitored over time. In parallel, PTX3-silenced ONS-76 cells and their respective shNT controls were implanted into the flanks of NOD/Scid mice.

Tumors derived from DAOY PTX3-KO clones exhibited a pronounced reduction in growth compared with MOCK controls, as demonstrated by decreased tumor volume and final tumor weight (Figure 3A and Supplementary Figure S9A). A similar inhibitory effect was observed *in vivo* using PTX3-silenced ONS-76 cells, which showed significantly reduced tumor volume and weight (Figure 3B and Supplementary Figure S9B) relative to shNT controls.

**Figure 3.**
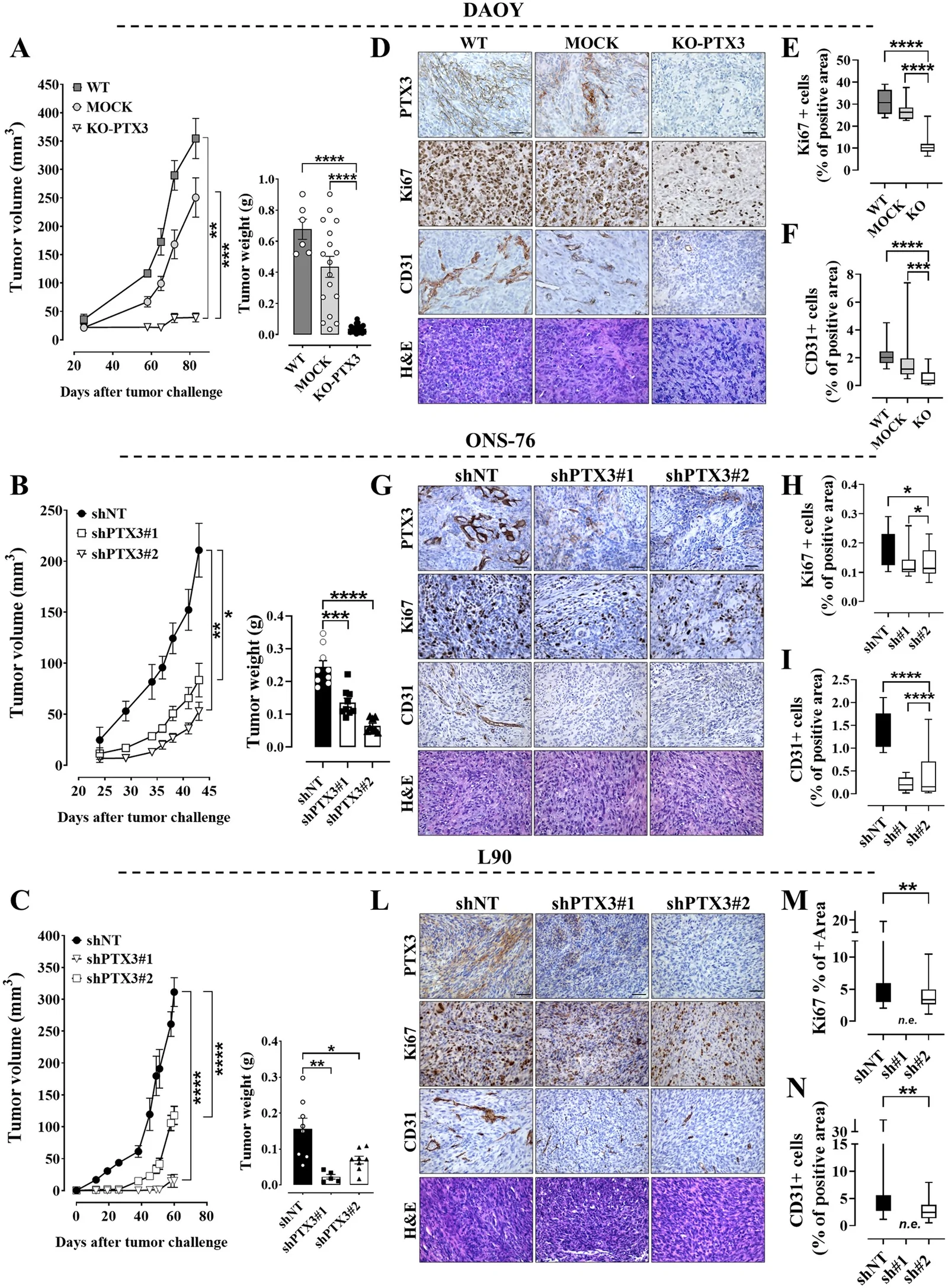
PTX3 downregulation and KO impair SHH-MB growth *in vivo*. Tumor growth and weight (A) of wild type cell population (WT), control/MOCK and PTX3-KO DAOY cells implanted subcutaneously in immune-compromised mice. Tumor growth and weight (B) of control (shNT) and PTX3-silenced (shPTX3#1-#2) of ONS-76 subcutaneous tumors. Tumor growth and weight (C) of control (shNT) and PTX3-silenced (shPTX3#1-#2) of L90 subcutaneous tumors. Representative pictures (D, G, L) and quantification of Ki67^+^ cells (E, H, M) and CD31^+^ area (F, I, N) of immunohistochemical analyses performed by ImageJ analysis on explanted samples. Scale bar: 50 μm. *N* = 6-10 mice/group. Data are the mean ± SEM; *P < .05, **P < .01, ***P < .005, ****P < .001. n.e = not evaluable (due to the small size and number of samples recovered).

Given the recognized limitations of human SHH-MB cell lines in recapitulating the original tumor phenotype, we further strengthened the translational relevance of our findings employing a murine cell line (L90 cells) derived from a murine *Ptch1*-deficient SHH-MB model.^39^ Notably, PTX3 silencing in L90 cells significantly reduced their proliferative capacity *in vitro* (Supplementary Figure S10) and markedly impaired tumor growth *in vivo* (Figure 3C and Supplementary Figure S9C), thereby confirming the tumor-promoting role of PTX3 in a translationally relevant SHH-MB model.

Histopathological analysis of the explanted tumors revealed a marked reduction in the proliferative activity, as indicated by decreased proliferation index (Ki67⁺ cells) in PTX3-KO DAOY (Figure 3D and E) and in PTX3-silenced ONS-76 (Figure 3G and H) and L90 tumors (Figure 3L and M). Importantly, evaluation of the tumor vasculature demonstrated a substantial decrease in microvascular density, assessed by CD31 staining, in PTX3-KO DAOY lesions (Figure 3D-F) as well as in PTX3-silenced ONS-76 (Figure 3G and I) and L90 (Figure 3L-N) tumors.

Taken together, *in vivo* data provides compelling evidence that PTX3 expression enhances SHH-MB tumorigenicity, supporting both tumor growth and angiogenesis.

### PTX3 Modulation Impacts the Angiogenic Potential of SHH-MB Cells

Prompted by the *in vivo* observation that PTX3 down-modulation markedly reduces tumor vascularization, we further investigated the contribution of PTX3 to SHH-MB–mediated angiogenesis using dedicated *in vitro* and *in vivo* assays.

We first evaluated the angiogenic potential of PTX3-silenced DAOY cells in a 3D endothelial sprouting assay using human umbilical vein endothelial cells (HUVECs). HUVEC spheroids co-cultured with shNT DAOY cells exhibited robust endothelial sprouting, whereas spheroids exposed to PTX3-silenced cells showed a significant reduction in sprouts’ number and length (Figure 4A), indicating a diminished ability of these cells to activate endothelial cells.

**Figure 4.**
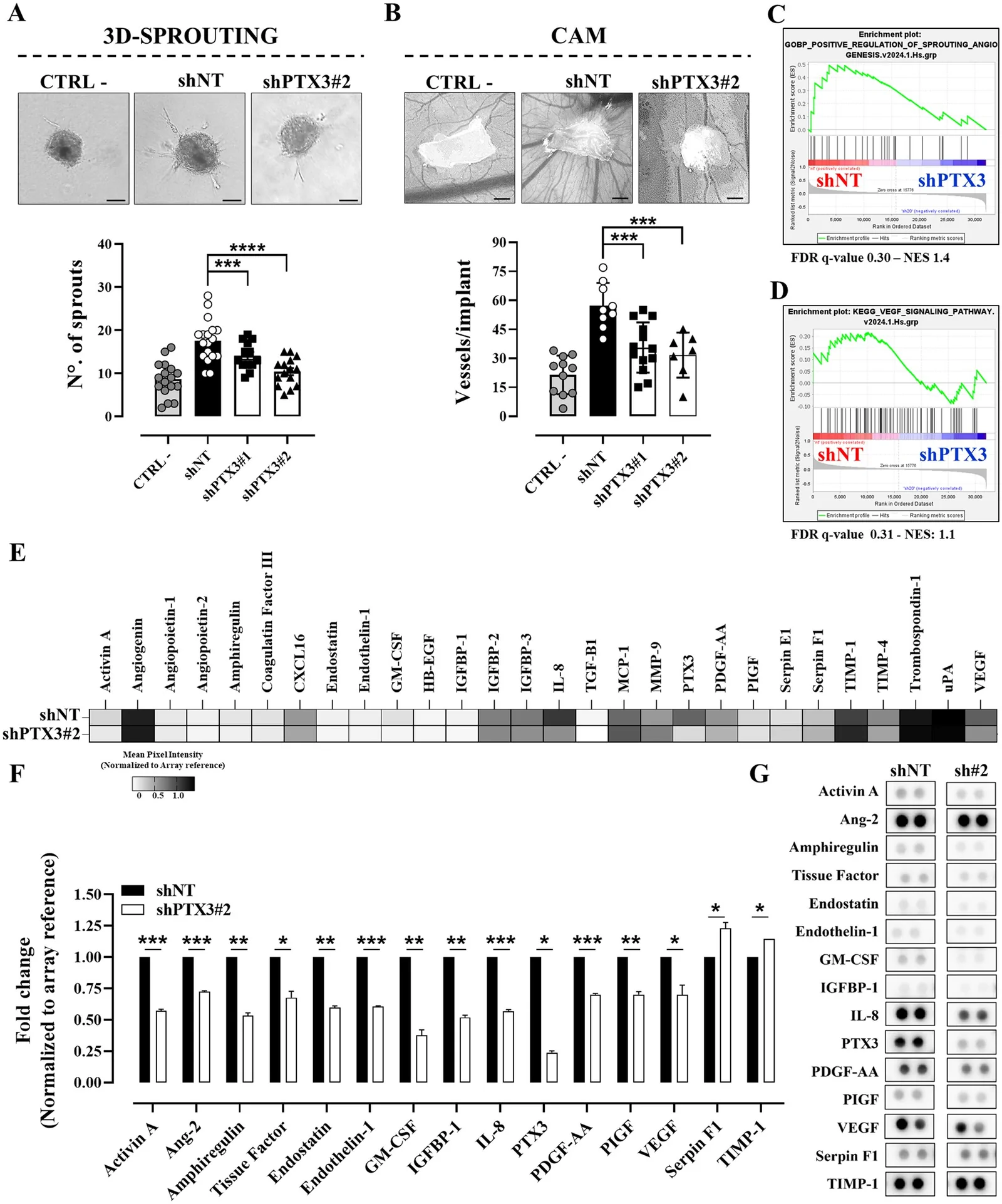
Effect of PTX3 modulation on the angiogenic potential of SHH-MB cells. (A) Representative pictures and quantification of 3D HUVEC spheroids embedded in fibrin gel, together with no cells (CTRL-), PTX3-silenced (shPTX3) or control (shNT) DAOY cells; scale bar 200μm. (B) Representative picture and quantification of the angiogenic response in chick embryo chorioallantoic membrane (CAM) induced by control (shNT), silenced (shPTX3) DAOY cells or no cells (CTRL-); scale bar 500μm. Gene Set Enrichment Analysis (GSEA) of genes associated with angiogenesis (C) and VEGF pathway (D) in shNT and shPTX3 DAOY cells. (E-G) Production of angiogenesis-related proteins detected by protein angioarray in shNT and shPTX3 DAOY cells. Data are the mean ± SEM; *P < .05, **P < .01, ***P < .005, ****P < .001.

To assess the angiogenic activity *in vivo*, we employed the CAM assay,^41^ which measures the capacity of tumor cells to induce neovascularization when seeded on the CAM surface of fertilized eggs. Both PTX3-silenced DAOY cells (Figure 4B) and PTX3-KO DAOY clones (Supplementary Figure S11) elicited a significantly attenuated angiogenic response, confirming a strong PTX3-dependent pro-angiogenic function *in vivo*.

Notably, the impaired angiogenic activity observed *in vitro* and *in vivo* was consistent with transcriptomic changes detected by GSEA. Indeed, PTX3 down-modulation was associated with significant negative enrichment of gene programs linked to angiogenesis and VEGF-A signaling (Figure 4C and D), suggesting a coordinated suppression of pro-angiogenic pathways.

To further characterize the molecular mechanisms underlying this phenotype, we analyzed angiogenesis-related protein secretion using a human angiogenesis profiler array. PTX3 silencing resulted in a broad reduction of key pro-angiogenic mediators, including VEGF-A, IL-8, and Angiopoietin-2, along with a concomitant increase in anti-angiogenic factors such as Serpin F1 and TIMP-1 (Figure 4E-G).

Together, these findings demonstrate that PTX3 strongly influences the angiogenic properties of SHH-MB cells, supporting its role as a pro-oncogenic regulator within this subgroup.

### PTX3 Activates TLR4/IRAK1/GSK-3/β-Catenin Signaling in SHH-MB Cells

To elucidate the molecular mechanisms through which PTX3 promotes SHH-MB tumor progression, we profiled the activation status of multiple kinases using a human phospho-kinase array. This analysis revealed differential phosphorylation of several proteins between control (shNT) and PTX3-silenced (shPTX3) DAOY cells, identifying modulation of key components of the TLR4 signaling cascade, including JNK, pSrc, PLCγ1, GSK-3, and β-Catenin^42-44^ (Figure 5A and Supplementary Figure S12).

**Figure 5.**
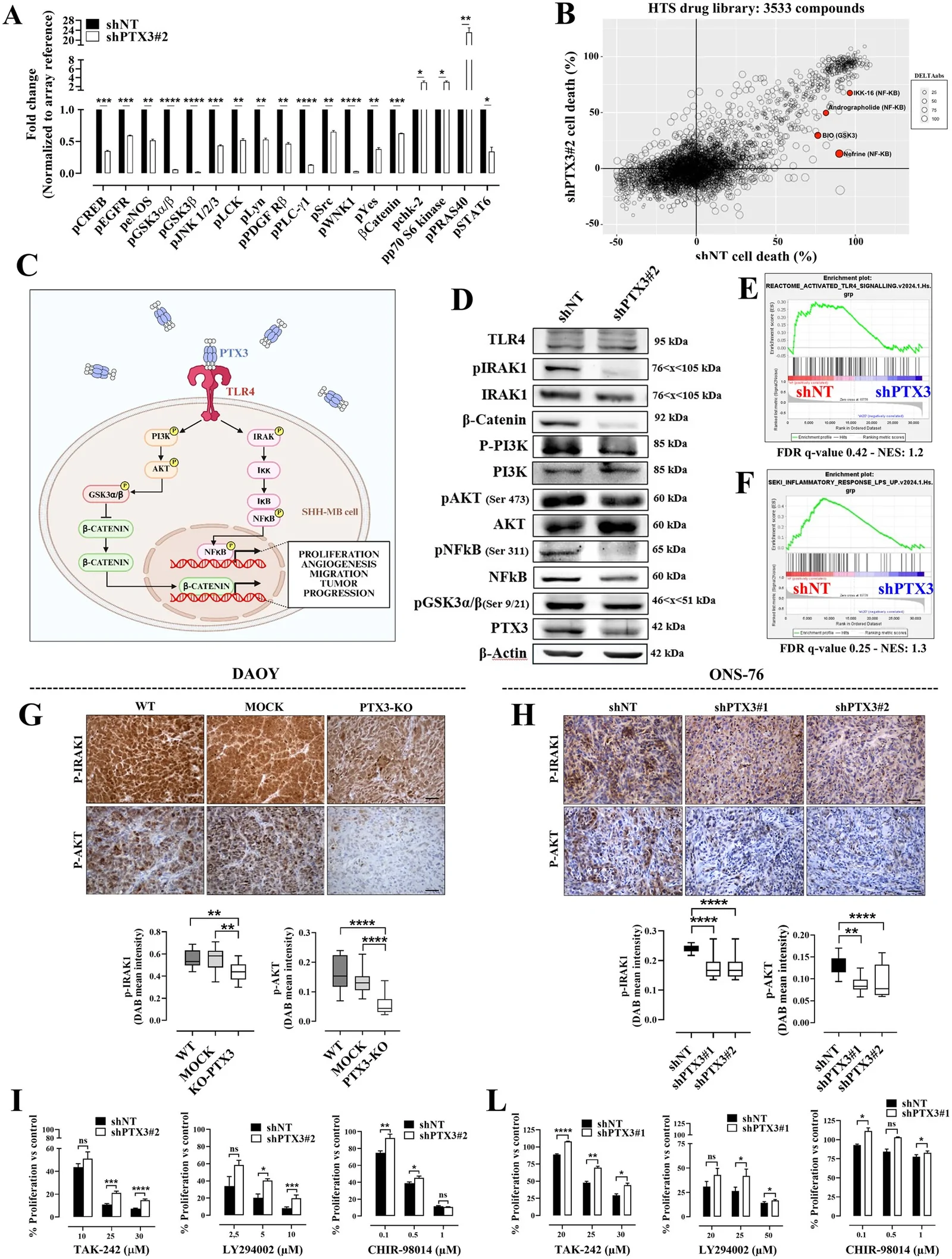
PTX3 activates TLR4/IRAK1/GSK-3αβ/β-catenin signaling in SHH-MB cells. (A) Human phospho-kinase array performed on PTX3-silenced and controls (shNT) DAOY cells. (B) Representative hits of the high-throughput drug screening performed on DAOY shNT and shPTX3 cells treated with a library of 3533 compounds. (C) Schematic representation of the TLR4/IRAK1/GSK-3/β-catenin signaling activated by PTX3 in SHH-MB (Created in BioRender. Ronca, R. [2025] https://BioRender.com/ m1xda8k). (D) Western blot analyses of DAOY shNT and shPTX3 cells of several TLR4 signaling mediators. Gene Set Enrichment Analysis (GSEA) of genes associated with inflammatory response to LPS (E) and TLR4 signaling (F) performed on DAOY (shNT *vs* shPTX3) cells. (G) Immunohistochemical analyses of DAOY controls (WT and MOCK) and PTX3-KO tumors, with representative pictures and quantification of pIRAK1 and pAkt; scale bar: 50 μm. (H) Immunohistochemical analysis of ONS-76 shNT and shPTX3 tumors with representative pictures and quantification of pIRAK1 and pAkt; scale bar: 50 μm. Antiproliferative assay preformed on silenced (shPTX3) and control (shNT) DAOY (I) and ONS-76 (L) cells with specific inhibitors TAK-242, LY294002, and CHIR-98014. Data are the mean ± SEM; *P < .05, **P < .01, ***P < .005, ****P < .001, ns = not significant.

In parallel, to functionally assess pathway dependencies, we conducted a high-throughput drug screening (HTS) using a 3,533-compound library, including 1,280 FDA-approved agents,^40^ on DAOY shNT and shPTX3 cells. Interestingly, in line with kinase profiling results, HTS screening revealed that PTX3-expressing DAOY cells were more sensitive to inhibitors of GSK-3 and NF-κB pathways (Figure 5B and Supplementary Figure S13). Indeed, these results are consistent with previous findings reporting the pathways downstream of PTX3-TLR4 interaction in triple-negative breast cancer and melanoma.^29^^,^^45^

As schematically summarized in Figure 5C and validated by Western blotting (Figure 5D), PTX3 activates TLR4 and induces 2 converging oncogenic pathways: IRAK1/NF-κB signaling and PI3K/Akt activation, which in turn inhibit GSK-3α/β, stabilizing β-catenin and promoting its nuclear translocation. PTX3 down-modulation resulted in a significant reduction of TLR4 pathway activation, as evidenced by decreased phosphorylation of IRAK1, Akt, PI3K, and NF-κB (p65) in shPTX3 DAOY cells compared with shNT controls (Figure 5D). Similar results were observed in ONS-76, UW-228 and L90 cells (Supplementary Figure S14A-C).

To further validate the specificity of the PTX3/TLR4 signaling axis, rescue experiments were performed by treating PTX3-silenced DAOY cells with recombinant PTX3 (recPTX3) protein. Indeed, recPTX3 treatment significantly restored proliferative capacity to levels comparable to shNT controls and reactivated downstream signaling (Supplementary Figure S15A and B). Moreover, blockade of PTX3 or TLR4 using ­specific neutralizing antibodies markedly reduced TLR4 pathway activation in control/DAOY shNT cells, reaching levels comparable to those observed in shPTX3 cells (Supplementary Figure S15C), and further supporting the role of PTX3 as an upstream regulator of TLR4 signaling in SHH-MB cells.

At the transcriptional level, GSEA revealed a trend toward negative enrichment of the TLR4-associated signaling program (Figure 5E), whereas a significant negative enrichment was observed (Figure 5F), further supporting attenuation of TLR4 signaling following PTX3 silencing.

Consistently, immunohistochemical analysis of explanted DAOY and ONS-76 tumors showed significantly reduced levels of activated IRAK1 and Akt in PTX3-silenced or PTX3-KO tumors compared to their respective controls (Figure 5G and H). Moreover, immunohistochemical analysis performed on a MB patients’ cohort further supported these findings, showing increased phospho-IRAK1 staining in the SHH subgroup compared with the other molecular subgroups (Supplementary Figure S16). This observation is consistent with the higher PTX3 expression levels detected in SHH-MB and further supports the activation of the PTX3-TLR4 signaling axis in this subgroup.

Based on these results, a TLR4 inhibitor (TAK-242) and two drugs targeting the downstream mediators of the TLR4 pathway ie, CHIR-98014 (GSK-3α/β inhibitor) and LY294002 (PI3K inhibitor), were selected for additional validation *in vitro*. As shown in Figure 5I-L  **(**and Supplementary Figures S17 and S18), these inhibitors were significantly more effective in high-PTX3-expressing (shNT) DAOY, ONS-76, and UW-228 cells than in their PTX3-depleted counterparts, further reinforcing the role of PTX3 in sustaining TLR4/IRAK1/GSK-3/β-catenin pathway activation.

Collectively, these results identify the PTX3/TLR4 as a functional upstream regulator of TLR4 signaling in SHH-MB and reveal a link between PTX3 expression and activation of converging oncogenic pathways controlling proliferation, survival, and β-catenin stabilization.

### Blockade of the PTX3/TLR4/IRAK1 Pathway Improves Chemotherapy Effect in SHH-MB

To strengthen the translational relevance of our mechanistic findings on the PTX3-TLR4 axis in SHH-MB, we next assessed whether inhibition of this pathway could potentiate the efficacy of the standard chemotherapeutic regimen for MB, consisting of Vincristine, Cisplatin, and Cyclophosphamide (VCC).^40^ Guided by *in vitro* data (Figure 5I-L and Supplementary Figures S17A and S18A), its upstream effect on TLR4 axis, and its demonstrated capacity to cross the blood-brain barrier (BBB),^46^ we selected TAK-242 as a prototypical and clinically relevant TLR4 inhibitor for combination studies.

As shown in Figure 6A and B, treatment with decreasing doses of VCC in combination with graded concentrations of TAK-242 resulted in a marked enhancement of the anti-tumor response. Notably, the interaction profile ranged from an additive effect in DAOY cells (Bliss synergy score: −1.56 ± 4.1; Figure 6A) to a clearly synergistic response in ONS-76 cells (Bliss synergy score: 16.41 ± 6.2; Figure 6B).

**Figure 6.**
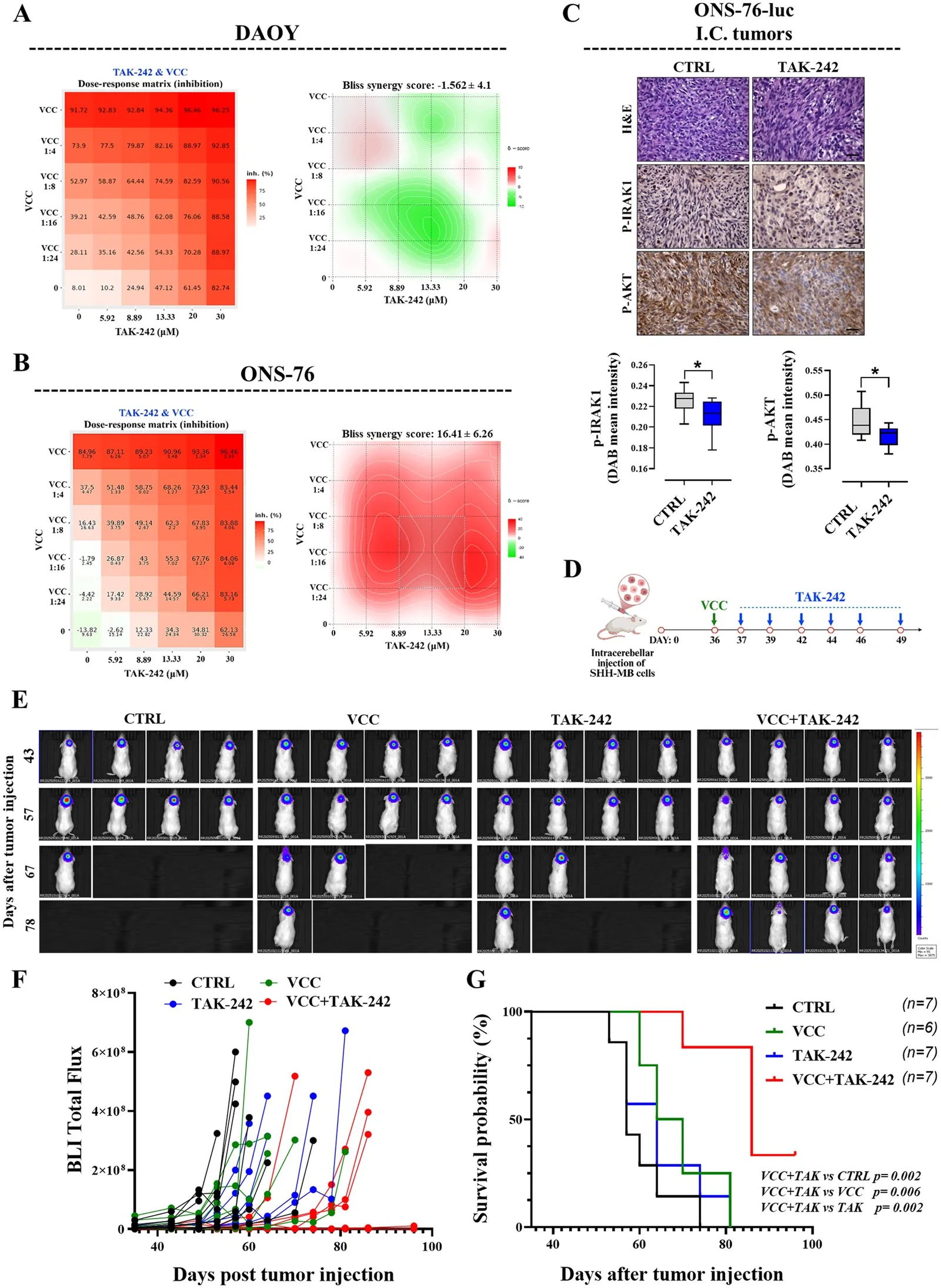
TLR4 inhibition in combination with chemotherapy increases survival of SHH-MB. (A) Inhibition of DAOY and (B) ONS-76 cell proliferation after combination treatment with increasing doses of TAK-242 and VCC. The Bliss Synergy Score (BSS) was calculated using Synergy Finder Plus. BSS > 10, BSS -10< × < 10, and BSS<-10 indicate synergism, additivity, and antagonism, respectively. (C) Immunohistochemical analyses of ONS-76-luc orthotopic tumors, after i.p. treatment with TAK-242 for 3 consecutive days, with representative pictures and quantification of pIRAK1 and pAkt; scale bar: 50 μm. **(**D) Schematic illustration of long-term survival study (Created in BioRender. Ronca, R. (2026) https://BioRender.com/ m1xda8k). (E) Representative bioluminescence images and (F) BLI-monitored growth of ONS-76-*luc* orthotopic tumors. (G) Kaplan-Meier survival curves up to day 100 after intra-cerebellar tumor challenge. *N* = 6/7 mice/group. Data are the mean ± SEM; *P < .05.

Based on these *in vitro* findings, we next investigated the therapeutic relevance of TLR4 inhibition *in vivo*, first assessing whether TAK-242 could attenuate TLR4 pathway activation in an orthotopic SHH-MB model. ONS-76 luciferase-expressing cells (ONS-76-*luc*) were implanted into the cerebellum of immunodeficient mice, and animals were treated with TAK-242 (3 mg/kg) for three consecutive days. As shown in Figure 6C, acute TAK-242 treatment significantly reduced the activation of phospho-IRAK1 and phospho-Akt within the tumor, confirming effective inhibition of TLR4 signaling *in vivo*.

Finally, we conducted a long-term survival study implanting intra-cerebellum ONS-76-*luc* cells and following longitudinally tumor evolution. After tumor take was established based on luciferase signal intensity at the implantation site, animals were assigned to receive treatments with vehicle, VCC, TAK-242, or the combination of VCC + TAK-242 (Figure 6D). Representative BLI images and quantitative analyses demonstrated that monotherapies produced a mild delay in tumor growth with no significant survival benefit (Figure 6E-G). In contrast, the combination of VCC with TAK-242 resulted in a marked improvement in survival, up to 100 days post tumor implantation, when compared with single treatments (Figure 6E-G).

Collectively, these data demonstrate that TLR4 inhibition potentiates the efficacy of standard chemotherapy in SHH-MB and suggest that targeting the PTX3/TLR4 axis may represent a promising therapeutic strategy for the SHH subgroup.

## Discussion

Medulloblastoma remains the most common malignant pediatric brain tumor, and despite substantial therapeutic advances, patients continue to experience significant treatment-related morbidity and variable survival outcomes across molecular subgroups. Among these, SHH-driven MB represents a biologically and clinically distinct entity, characterized by aberrant activation of Sonic Hedgehog signaling, marked heterogeneity, and, in certain age groups, poor prognosis.

In this context, our study reveals PTX3 as a subgroup-enriched mediator of SHH-MB biology and aggressiveness. We demonstrate that PTX3 is highly expressed in SHH-MB patients’ samples and cell lines, compared to both normal cerebellum and other MB subtypes. This distinct expression profile prompted functional investigations, through which we show that PTX3 critically contributes to multiple malignant features, including cell proliferation, migration, clonogenicity, and angiogenesis. Indeed, loss-of-function approaches consistently impaired these tumorigenic functions and significantly reduced tumor formation and vascularization *in vivo*, thus underscoring the biological relevance of PTX3 within the SHH-MB molecular framework.

Despite the known limitations of available human SHH-MB models/cell lines, we further strengthened our findings by validating them in a translationally relevant murine model (*Ptch1*-deficient mouse-derived cells).^39^ Importantly, PTX3 silencing recapitulated the observed phenotypes, reducing tumorigenic potential both *in vitro* and *in vivo*, thereby reinforcing the robustness and translational relevance of our results.

Mechanistic analyses revealed that PTX3 exerts its pro-tumorigenic effects by activating a complex TLR4/IRAK1/PI3K-Akt/GSK-3/β-catenin signaling cascade, a pathway known to integrate neuro-inflammatory cues with proliferative and survival programs. The identification of this axis is particularly noteworthy, as SHH-MB progression is increasingly recognized to be shaped not only by canonical SHH pathway alterations but also by additional modulators of tumor microenvironment, inflammation, and cell-cell communication.^47^ Our findings place PTX3 at the inter­section of these processes, functioning as an upstream amplifier of TLR4 activity and promoting downstream stabilization of β-catenin, a key effector implicated in many tumor features. These observations provide a mechanistic rationale for considering PTX3-TLR4 signaling as a relevant player of SHH-MB biology.

The translational impact is reinforced by the fact that pharmacologic inhibition of TLR4 using TAK-242 attenuated downstream signaling in orthotopic tumors and produced an additive to synergistic response in SHH-MB cells when combined with standard multi-agent chemotherapy. Most importantly, the TAK-242/chemotherapy (VCC) combination significantly extended survival *in vivo*, whereas single-agent treatments did not yield meaningful benefit. This synergy suggests that PTX3 or TLR4 blockades may enhance the vulnerability of SHH-MB cells to cytotoxic agents, potentially enabling more effective therapeutic regimens or future strategies aimed at reducing treatment intensity and long-term side effects. In fact, one of the most critical challenges is the limited penetration of drugs across the BBB (ClinicalTrials.gov, NCT01878617). Indeed, evidence shows that the BBB remains intact in SHH-MB, thus limiting the effectiveness of agents in treating these tumors.^48^^,^^49^ In this context, TAK-242 represents a promising drug since it has been demonstrated to successfully cross the BBB, highlighting its potential as an effective therapeutic option for targeting brain tumors.^46^^,^^50^

Collectively, our findings position PTX3 as both a mechanistic driver and a promising therapeutic node in SHH-MB paving the way for targeting PTX3 or its downstream signaling partners as a novel avenue for improving the treatment landscape for patients with SHH-MB, a subgroup in urgent need of more effective and less toxic therapies.

## Supplementary Material

noag167_Supplementary_Data

## Data Availability

All data are available.
