# Supplementary material for: A PTX3/TLR4 axis sustains sonic hedgehog-medulloblastoma growth and defines a new therapeutic vulnerability
Source: Neuro Oncol. 2026 Jul 23;28(11):2684–99. doi: 10.1093/neuonc/noag167 (PMC13731783; doi:10.1093/neuonc/noag167)
Supplement: Supplementary file 1 [file noag167_supplementary_data.docx]

**SUPPLEMENTARY DATA**

**Figure S1. In silico analysis of PTX3 expression in MB and normal cerebellum by R2 Genomics Analysis and Visualization Platform. A**) Across Datasets Analysis of PTX3 expression in normal cerebellum and medulloblastoma (Roth and Pfiser datasets). **B**) PTX3 expression in normal cerebellum and medulloblastoma in Pomeroy dataset. **C-D**) PTX3 expression in MB subgroups in Swartling and Northcott datasets.

|  |  | **Upregulated genes** | **PTX3 ranking** | **logFC** (*PTX3*) |
| --- | --- | --- | --- | --- |
| **SHH** | *vs* **Group 3** | 782 | 142 | 2.1 |
|  | *vs* **Group 4** | 765 | 381 | 1.4 |
|  | *vs* **WNT** | 709 | 107 | 2.3 |

**Figure S2.** PTX3 ranking among the upregulated genes in SHH MB samples compared with other MB subgroups. Analysis was performed using Cavalli dataset and upregulated genes were selected with pvalue ≤0.05 and logFC ≥1.

**Figure S3.** QuPath‑based quantification of PTX3 staining performed in Figure 1B-C. Data are the mean±SEM; *p<0.05, ***p<0.005, ****p<0.001.

**Figure S4. PTX3 silencing validation in SHH-MB cell lines and it affects on SHH-MB cells.** **A**-**B**) PTX3 silencing in DAOY cell line was verified by ELISA and RT-qPCR. **C**)PTX3 silencing was verified by RT-qPCR in ONS-76 (**C**) and UW-228 (**D**) cell lines. Data are the mean±SEM; *p<0.05, **p<0.01, ***p<0.005, ****p<0.001.

**Figure S5.** Gene Set Enrichment Analysis (GSEA) in DAOY silenced cells (shPTX3) and control (shNT) of genes associated with proliferation and migration.

**B**

**A**

**Figure S6. Characterization of PTX3 knock-out clones of DAOY cells using CRISP/Cas9 technology. A)** PTX3 expression levels determined by ELISA in DAOY wild type (WT), MOCK and KO clones. n.d.= not detectable. **B**) PTX3 localization on chromosome 3, representation of exons, and illustrations of SANGER and NGS for KO of PTX3 clones compared to the reference sequence. Depiction of protein prediction analysis for the mutated sequence by Expasy.

**Figure S7.** Representative pictures of wound healing (A) and colony assay (B) performed on DAOY cells and quantified in Figure 2M-N respectively.

**Figure S8. PTX3 silencing does not affect proliferation in Group 3 MB cells. A**) Western blot analysis showing PTX3 silencing by siRNA in HD-MB03 cells. HSP70 was used as loading control.

**B**) Cell proliferation analysis of control and PTX3-silenced HDMB03 cells. Data are the mean±SEM; ns= not significant.

**Figure S9.** Representative pictures of tumors explanted from DAOY (**A**), ONS-76 (**B**), and L90 (**C**) xenografts reported in Figure 3.

**Figure S10. PTX3 silencing impairs growth of murine L90 cells. A)** Western blot analysis showing PTX3 silencing in the murine L90 cell line (Ptch1^+/-^ p53^-/-^). **B**) Cell proliferation analysis of control (shNT) and PTX3-silenced L90 cells (shPTX#1 and shPTX3#2) at 72 hours. **C**) Representative images of neurosphere growth in control (shNT) and PTX3-silenced L90 cells. Data are the mean±SEM; *p<0.05; scale bar= 500µm.

**Figure S11. Effect of PTX3 modulation on the angiogenic potential of SHH-MB cells.** The knock-out clones of PTX3 reduced the angiogenic response in chick embryo chorioallantoic membrane (CAM) compared to control ones. Scale bar 500μm. Representative picture and quantification are reported. N= 8-10 eggs/point; Data are the mean±SEM; ****p<0.001.

**Figure S12.** Human phospho-kinase array performed on PTX3-silenced and controls (shNT) DAOY cells and quantified in Figure 5A.

**Figure S13. HTS plate quality control.** Dot plot summarizing the distribution of Z’ factor values calculated for each test plate screened within our study. The Z-prime (Z’) quality control metrics was calculated for each plate throughout the entire HTS to measure the separation between the positive control (Benzethonium Chloride 75μM) and negative control (0.5% DMSO) after 72 hours of drug exposure. For this screening, only plates with acceptable quality metrics (Z’ > 0.4) were further analyzed.

**A**

**B**

**C**

**Figure S14. PTX3 activates TLR4/IRAK1/GSK-3/β-catenin signaling in SHH-MB cells. A**) Western blot analysis of TLR4 downstream signaling pathway mediators evaluated in ONS-76 shNT and shPTX3 (**A**), UW-228 shNT and shPTX3 (**B**), and L90 shNT and shPTX3 (**C**) cells.

**A**

**B**

**C**

**Figure S15. Rescue of PTX3/TLR4 signaling in PTX3-silenced DAOY cells by recombinant PTX3. A)** Cell proliferation analysis at 72 hours in DAOY shNT and shPTX3 cells treated or not with recPTX3 (100 nM). **B)** Western blot analysis performed in DAOY shNT and PTX3-silenced cells treated or not with recombinant PTX3 protein (recPTX3). Phospho-IRAK1 and phospho-NF-κB levels were evaluated to assess reactivation of the PTX3/TLR4 signaling pathway. **C**) Western blot analysis for the activation of TLR4 signalling pathway, performed on DAOY shNT and shPTX3 cells treated or not with anti-PTX3 or anti-TLR4 antibodies (10 μg/ml).

**Figure S16. IRAK1 activation is enriched in the SHH MB.** Immunohistochemical analysis of phospho-IRAK1 performed on our MB patient cohort (as described in Figure 1B-C). Representative images of phospho-IRAK1 staining across the four molecular subgroups are shown together with the corresponding quantification of staining intensity among subgroups. Scale bar= 50µm; data are the mean±SEM; **p<0.01, ****p<0.001.

**A**

**B**

**C**

**Figure S17.** Inhibition of DAOY shNT and shPTX3 colonies formation after treatment with (**A**) TAK-242 (30μM), (**B**) LY294002 (5μM), and (**C**) CHIR-98014 (0.5μM). The graphs represent the absorbance after crystal violet staining and solubilization of the colonies. Data are the mean±SEM; *p<0.05, **p<0.01, ****p<0.001

**A	B	C**

**Figure S18. PTX3 activates TLR4/IRAK1/GSK-3/β-catenin signaling in SHH-MB cells.** Inhibition of UW-228 shNT and shPTX3 cell proliferation after treatment with increasing doses of (**A**) TAK-242, (**B**) LY294002, and (**C**) CHIR-98014. Data are the mean±SEM; *p<0.05, **p<0.01, ***p<0.005, ns= not significant.
